# Targeting Secondary Hematoma Expansion in Spontaneous Intracerebral Hemorrhage – State of the Art

**DOI:** 10.3389/fneur.2016.00187

**Published:** 2016-10-25

**Authors:** Jian Guan, Gregory W. J. Hawryluk

**Affiliations:** ^1^Department of Neurosurgery, Clinical Neurosciences Center, University of Utah, Salt Lake City, UT, USA

**Keywords:** spontaneous intracerebral hemorrhage, management, animal models, therapeutic targets, translational research, secondary injury, hematoma

## Abstract

Spontaneous intracerebral hemorrhage (SICH), defined broadly as intracerebral hemorrhage not related to trauma, results in long-term disability or death in a large proportion of afflicted patients. Current management of this disease is predominantly supportive, including airway protection, optimization of hemodynamic parameters, and management of intracranial pressure. No active treatments that demonstrate beneficial effects on clinical outcome are currently available. Animal models of SICH have allowed for the elucidation of multiple pathways that may be attractive therapeutic targets. A minority of these, such as aggressive blood pressure management and recombinant activated factor VII administration, have led to large-scale clinical trials. There remains a critical need for further translational research in the realm of SICH.

## Introduction

Spontaneous intracerebral hemorrhage (SICH), while comprising only 10–20% of all strokes (1, 2), remains one of the deadliest forms of the disease, with mortality rates approaching 40% at 1 month (3). Long-term survivors of SICH are often saddled with permanent deficits, with up to 75% of patients suffering significant disability or mortality at 1 year (4). Management of SICH patients currently consists primarily of supportive therapies (5), such as airway management, hemodynamic monitoring, and control of intracranial pressure (6), with no treatment options demonstrating significant efficacy despite extensive investigation into the topic (7).

Despite the disappointing results of interventional studies to date, there is reason to be hopeful going forward. Advancements in the understanding of secondary injury after SICH have highlighted opportunities for therapeutic intervention (5). One such opportunity is preventing secondary expansion of hemorrhage after the initial bleed. Such expansion may occur in up to 30% of SICH patients (8, 9) and is associated with significantly worse clinical outcomes (10). This impact on outcome is independent of previously described predictors of outcome in SICH (11), including patient age, Glasgow Coma Scale score, intraventricular extension, hematoma volume, hemorrhage location, anticoagulant use, and medical history (12–14).

This review will discuss the classifications and current animal models of SICH, as well as what is known about the pathophysiology of secondary hematoma expansion. The interaction between bench research and clinical trials will be examined, with a focus on blood pressure control and the hemostatic mechanism – two areas where findings in animal models of SICH have lead to large-scale, randomized controlled trials in humans.

## SICH Etiology

Broadly, SICH is defined as any intracerebral hemorrhage that is non-traumatic in nature; SICH can be further divided into primary and secondary hemorrhage (15). Primary SICH consists of those hemorrhages in which an underlying vascular malformation or coagulopathy is not identified (16). The two most common causes of primary SICH are arteriosclerosis due to chronic hypertension and cerebral amyloid angiopathy, which together account for up to 88% of all primary SICH (17). Chronic hypertension initially leads to proliferation of smooth muscle cells in the small penetrating arterioles of the brain, but eventually smooth muscle cell death occurs, with replacement of muscle in the tunica media layer with collagen (18). This weakening of the arteriolar wall can lead to vessel ectasia – Charcot–Bouchard aneurysms – and subsequent rupture; it occurs primarily in the deep, penetrating arterioles of the brain (19). In cerebral amyloid angiopathy, the progressive deposition of insoluble amyloid protein in the walls of small- and medium-sized vessels leads to increased vessel fragility over time (20). This deposition increases dramatically with age and occurs primarily in the leptomeningeal and cortical vasculature (21). As a result, SICH caused by cerebral amyloid angiopathy is significantly more common in the elderly population and is more commonly seen in a superficial cortical distribution (21). Patients with cerebral amyloid angiopathy are also at higher risk of recurrent hemorrhage (22).

Secondary SICH can be caused by a variety of underlying lesions and pathologies. Vascular malformations that can lead to SICH include arteriovenous malformations (23), cerebral aneurysms (24), dural arteriovenous fistulas (25), and cavernous malformations (26). Patients who have had ischemic strokes can experience hemorrhagic conversion (27), as can up to 50% of cerebral venous thrombosis patients (28). Neoplastic causes of SICH make up a minority of cases, but melanoma, choriocarcinoma, renal cell carcinoma, and thyroid carcinoma are the most prone to bleeding (29). Investigations into secondary hematoma expansion in secondary SICH are fairly limited, with the exception of those evaluating hemorrhages associated with oral anticoagulant use, where secondary expansion is both more common and associated with worse outcomes (30).

## Animal Models

Currently, there are two widely used paradigms for modeling of SICH in animals. The first is the intracerebral injection of autologous blood. Initially developed in the 1960s (31), this model has been used in both large animals (32–34) (with injections typically performed into the frontal lobe) and rodents (15, 35) (with injections typically performed into the basal ganglia). This model has the advantage of allowing for control of hemorrhage volume but does not mimic the effects of vessel rupture seen in SICH (36). The second model involves injection of collagenase into the brain, leading to compromise of the extracellular matrix and subsequent vessel rupture. Developed in the 1990s and used primarily in rodents (37, 38), this model replicates the vascular disruption seen in SICH but has more diffuse effects when compared with the injection model and may result in diffuse inflammation and ischemia that is not commonly seen in the disease process (39). Despite these shortcomings, the collagenase injection model is more commonly used in the evaluation of secondary hematoma expansion.

## Pathophysiology of Hematoma Expansion

Although the precise mechanism for secondary hematoma expansion has yet to be fully elucidated, two predominant models currently exist. The first is the “persistent bleeding” model, which proposes that the vessel or vessels that initially rupture continue to bleed and lead to an enlarging hematoma (7). The second model, initially proposed by Fisher (40), postulates that secondary expansion results from the mechanical disruption of neighboring vasculature by the initial bleed.

**Figure 1:**
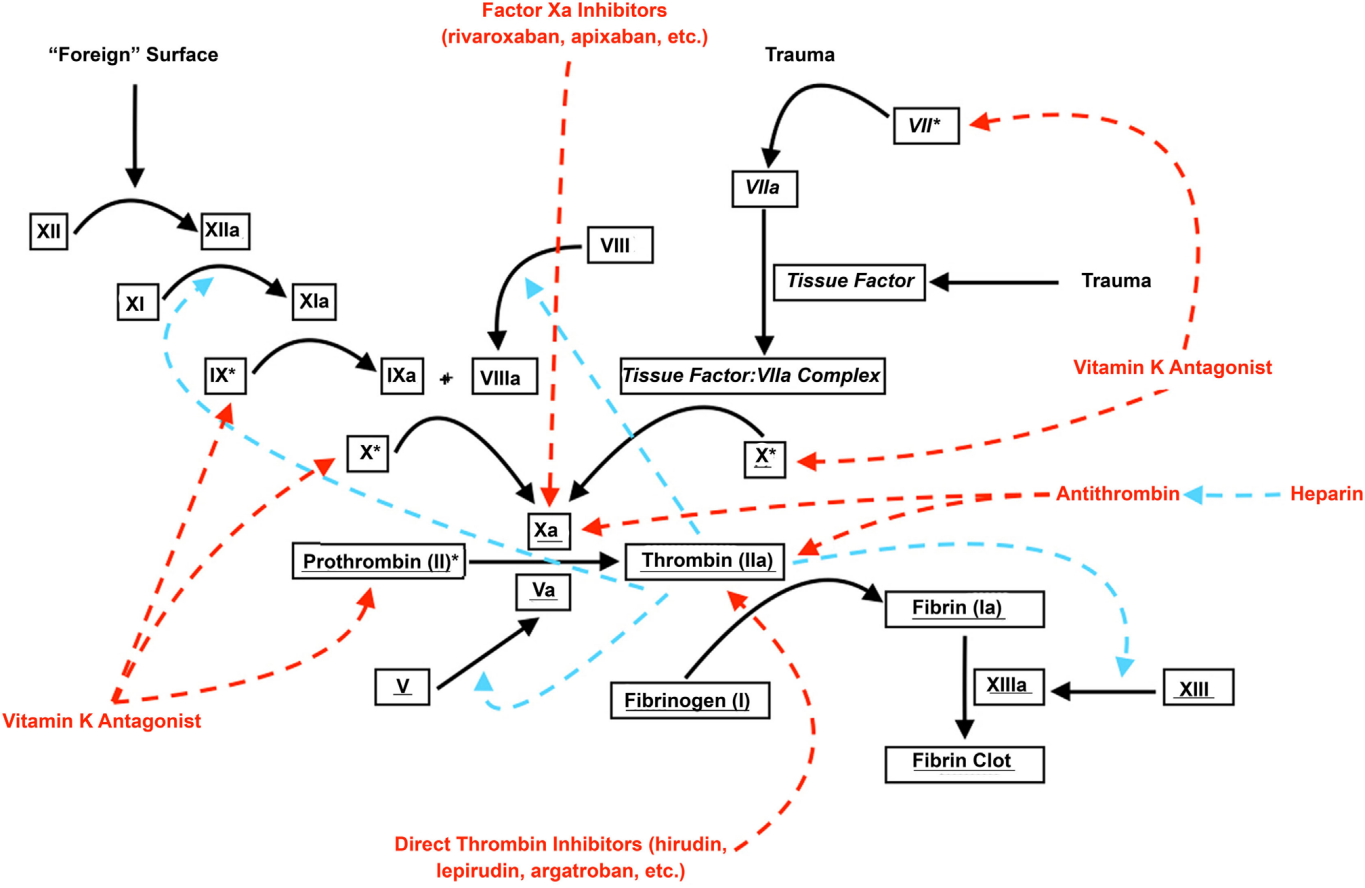
**Coagulation cascade and anticoagulants**. Roman font denotes parts of the “intrinsic” coagulation pathway; italicized font denotes parts of the “extrinsic” coagulation pathway; underlined font denotes parts of the “common” coagulation pathway. Dashed red arrows denote inhibition; dashed blue arrows denote augmentation. * denotes vitamin K-dependent coagulation factors.

Regardless of the underlying histopathological cause, experimental evidence suggests that two important contributors to secondary hematoma expansion may be derangement of the coagulation cascade (41, 42) (Figure 1) and hypertension. A variety of coagulopathies have been shown to be associated with an increased risk of secondary hematoma expansion. In a murine collagenase injection model of SICH, Illanes et al. (43) demonstrated significantly higher rates of hematoma expansion and larger hematoma volumes in animals treated with warfarin when compared with controls. A variety of newer anticoagulant agents act to inhibit either factor Xa (e.g., rivaroxaban) or thrombin (e.g., dabigatran). By doing so, these agents halt the so-called “thrombin burst,” whereby a single molecule of factor Xa may activate several hundred molecules of thrombin (44). This “thrombin burst” subsequently upregulates myriad other coagulation factors (45). For these novel agents, Zhou et al. (46) found that hematoma volume and secondary expansion were significantly increased in a subgroup of mice treated with rivaroxaban and high-concentration collagenase (47). Surprisingly, Lauer et al. (48) found that secondary hematoma expansion was not dramatically higher in animals treated with dabigatran compared with control animals, although larger hemorrhagic volumes and worsened neurologic function were seen with high-dose parenteral administration of the drug. The myriad disorders, which may affect the coagulation cascade, have led to the development of more refined laboratory testing methods to detect and track its function. One of the most widely adopted of these is thromboelastography (49), a modality that detects variables such as the rate of fibrin formation, the thrombin burst, and the firmness of the resultant clot (50). Such tests may allow for the stratification of patients at risk of hematoma enlargement and represents an area for future study (50).

Non-pharmacologic disruption of hemostatic mechanisms may be equally important. In an autologous blood injection rat model of intracerebral hemorrhage, Liu et al. (51) found a relationship between hyperglycemia and secondary hematoma expansion. They discovered that this relationship may be mediated by the platelet-inhibiting effects of plasma kallikrein, a protein whose action is enhanced by elevated blood glucose. Hyperglycemia has also been shown in animal models to worsen perihematomal neurolysis and edema, likely secondary to increased levels of inflammatory cytokines such as TNF-α (52). Current work on intensive hyperglycemic management in ischemic stroke is ongoing through the multicenter Stroke Hyperglycemia Insulin Network Effort (SHINE) (53), but organized efforts in hemorrhagic stroke are needed.

The relationship of hypertension to secondary hematoma expansion is more controversial. Although some experimental evidence does exist that higher blood pressures may lead to hematoma expansion and larger overall hematoma volumes (54), other investigators have found no relationship between hypertension and hematoma expansion (55). This variability may be related to the acuity of blood pressure rise, with large, rapid changes in blood pressure being less well tolerated than chronic hypertension (56). One rat model of hypertensive hemorrhagic stroke showed increased vascular permeability on MRI in hypertensive rats in the 1–2 weeks prior to SICH (57), suggesting that vessel changes may be detectable prior to catastrophic hemorrhage. Both animal (58) and human (27) studies also suggest that patients with anticoagulation and hypertension may be at further risk of intracerebral hemorrhage, and focused investigation on these commonly overlapping entities is also warranted.

## Translational Therapies

### Blood Pressure Control

Animal models have suggested that elevations in blood pressure may lead to increased secondary hematoma expansion in SICH. Several observational analyses in patients also suggested that hypertension was associated with worsened clinical outcomes following SICH and that aggressive blood pressure management could offer benefits (59, 60). This has led to the initiation of prospective, randomized trials seeking to determine the efficacy of aggressive blood pressure management in reducing secondary hematoma expansion and overall morbidity.

The Intensive Blood Pressure Reduction in Acute Cerebral Hemorrhage Trial (INTERACT) was initiated in 2005, with patients randomized to intensive (systolic blood pressure goal of <140 mmHg) or standard (systolic blood pressure goal of <180 mmHg) groups (4). Although early pilot data suggested that aggressive blood pressure reduction reduced secondary hematoma expansion (4), the results of the main study (INTERACT2) did not demonstrate significant reduction in secondary hematoma expansion with aggressive hypertension correction [relative difference 4.5%, 95% confidence interval (CI) – 3.1–12.7; *p* = 0.27] (61). INTERACT2 also did not find any significant difference between the two groups in the primary outcome of severe disability or death at 90 days [odds ratio (OR) 0.87 for the intensive treatment group; 95% CI 0.75–1.01; *p* = 0.06], although patients in the intensive blood pressure control group had a higher rate of good functional outcomes (OR 0.87, 95% CI 0.77–1.00; *p* = 0.04) on ordinal analysis of modified Rankin scores at 90 days.

Similarly, early results of the Antihypertensive Treatment of Acute Cerebral Hemorrhage (ATACH) study (62), where patients were treated using nicardipine for a systolic blood pressure goal of <140 mmHg, also did not find significant reductions in the rate of secondary hematoma expansion (21% in patients with lower than median systolic blood pressure reduction versus 31% in patients with greater than median systolic blood pressure reduction, *p* > 0.05), although a trend was noted. The follow-up study, ATACH-2, demonstrated no significant advantage of aggressive blood pressure control (with a goal of 110–139 mmHg) over standard (140–179 mmHg) and was discontinued after 1280 patients were enrolled (63). For the primary outcome of death or disability in ATACH-2, patients with intensive management had a rate of 37.7%, while those in the standard group carried a risk of 38.7% (relative risk 1.04; 95% CI 0.85–1.27). Secondary hematoma expansion occurred in 18.9% of patients in the intensive treatment group and 24.4% of the standard treatment group (*p* = 0.08). Patients in the intensive treatment group also suffered a significantly higher rate of short-term renal complications, with a rate of 1.6 versus 1.2% in the standard treatment group (*p* = 0.002).

One of the primary concerns regarding aggressive blood pressure management in intracerebral hemorrhage is the fear of reduced cerebral perfusion (64). The intracerebral hemorrhage Acutely Decreasing Arterial Pressure Trial (ADAPT) tested the effects of acutely lowering systolic blood pressure within 1 h of randomization to <150 mmHg in one group and <180 mmHg in another (65). The study found no difference in perihemorrhage cerebral blood flow volumes on CT perfusion between the two groups (0.86 ± 0.12 mL/100 g/min in the <150 mmHg group versus 0.89 ± 0.09 mL/100 g/min in the <180 mmHg group, *p* = 0.19). This led the investigators to conclude that aggressive blood pressure lowering in SICH patients did not lead to lowered cerebral blood flow or risk of perihemorrhagic ischemia. In light of the results of these studies, the most recent American Heart Association/American Stroke Association guidelines recommend an acute systolic blood pressure goal of <140 mmHg and a long-term goal of <130 mmHg to prevent recurrent SICH (66).

### Correction of Hemostatic Derangement

Derangements of the hemostatic mechanism have been demonstrated in animal models to significantly increase the risk of secondary hematoma expansion and overall hemorrhage volume. Patients presenting with intracerebral hemorrhage are more than three times as likely to be taking an anticoagulant when compared with matched controls and were significantly more likely to be utilizing antiplatelet agents (67, 68). Compared with patients who are not on anticoagulation therapy, those with SICH on blood-thinning agents have dramatically higher rates of poor outcome and mortality (69). Reversal of anticoagulation in animal models (70) has been shown to attenuate hematoma expansion and reduce final hematoma volume. However, despite the ubiquity of the anticoagulated SICH patient there remains no evidence from randomized trials evaluating the efficacy of anticoagulation reversal in SICH patients (71). Evidence from retrospective analyses of anticoagulation reversal suggests that even in patients who are effectively treated, hematoma expansion remains prevalent, and poor outcomes remain common (47). The majority of these studies have evaluated only patients who take warfarin for anticoagulation, and there is a significant lack of data on the behavior of more recent, novel anticoagulants. There is also a lack of evidence regarding the utility of newer anticoagulation reversal agents in the prevention of hematoma expansion in SICH. These include general-use agents, such as four-factor prothrombin complex concentrates (72) (trade name Kcentra, approved for use in the United States in 2013), and targeted drugs, such as the antibody idarucizumab (trade name Praxibind, approved for use in the United States in 2015), which inactivates dabigatran (73). As a result, although guidelines exist for the reversal of anticoagulation in SICH, the majority of these are good-practice recommendations without extensive, high-quality supporting evidence (74).

Although an ever-increasing number of patients presenting with SICH are on anticoagulant therapy, the majority of cases of SICH still occur in those taking no blood-thinning agents. The utility of prothrombotic agents in non-anticoagulated animals has been primarily focused on the utility of recombinant factor VII (rFVIIa), with rat models showing significantly reduced hematoma expansion in animals treated with rFVIIa compared with controls (75). The results of these studies led to the Factor seven for Acute hemorrhagic Stroke Trial (FAST), a randomized, controlled study evaluating the efficacy of rFVIIa in SICH. Although the phase II study showed significant reductions in hematoma expansion and improvements in clinical outcome (76), the phase III study failed to demonstrate any clinical benefit, with a similar rate of death or severe disability at 90 days in the low-dose rFVIIa group (26%), the high-dose rFVIIa group (30%), and the placebo group (24%, *p* > 0.05), although volumes of hematoma expansion remained lower in both rFVIIa groups compared with placebo (18% increase, *p* = 0.08 for the low-dose group and 11% increase, *p* < 0.001 for the high-dose group versus 26% increase for placebo) (77).

Some concern remained after these studies that patient selection – specifically the enrollment of individuals who were unlikely to suffer hematoma expansion in the first place – may have resulted in the negative results of FAST. Thus, two trials (the Selection of Intracerebral Hemorrhage to Guide Hemostatic Therapy or SPOTLIGHT and the Spot Sign for Predicting and Treating ICH Growth Study or STOP-IT) are ongoing to attempt to resolve this question. Both these studies utilize the so-called “spot sign” for selection of patients who are at higher risk of hematoma expansion. The spot sign is the presence of contrast enhancement within the hematoma mass on CT angiography and has been shown to be strongly associated with both hematoma expansion and poor outcome (78, 79). However, the sensitivity of the spot sign is low – only 51% in a recent prospective series, and a significant number of patients without contrast extravasation on CT angiography are still at risk of secondary hematoma growth (80).

## Conclusion

Because of the high mortality and incidence of long-term disability associated with SICH, it remains a significant health care burden globally. Current therapeutic options remain limited, but continued investigations into the underlying mechanisms of secondary injury, such as hemorrhage expansion, have yielded promising targets for intervention that continue to warrant further investigation. Further refinement of animal models of SICH, the discovery of therapeutic pathways in the laboratory setting, and the transition of this knowledge into treatments in humans remain critically needed.

## Author Contributions

JG and GH conceived of the topic, drafted the manuscript, and did critical review and revision of manuscript.

## Conflict of Interest Statement

The authors declare that the research was conducted in the absence of any commercial or financial relationships that could be construed as a potential conflict of interest. The reviewer JP and handling editor declared their shared affiliation, and the handling editor states that the process nevertheless met the standards of a fair and objective review.
